# OpenMSK: A modular open-source framework for comprehensive analysis of imaging biomarkers in knee osteoarthritis

**DOI:** 10.1016/j.ocarto.2026.100886

**Published:** 2026-10-09

**Authors:** Ananya Goyal, Francesca Belibi, Vyoma Sahani, Rune Pedersen, Yael Vainberg, Ashley Williams, Constance Chu, Bryan Haddock, Garry E. Gold, Akshay S. Chaudhari, Feliks Kogan, Anthony A. Gatti

**Affiliations:** a Department of Radiology, Stanford University, CA, USA; b Department of Bioengineering, Stanford University, CA, USA; c College of Osteopathic Medicine of the Pacific, Western University of Health Sciences, CA, USA; d Department of Clinical Physiology, Nuclear Medicine & PET, Rigshospitalet, Copenhagen University Hospital, Denmark; e Department of Orthopaedic Surgery, Stanford University, CA, USA

**Keywords:** Knee MRI, Automated image segmentation and analysis, Musculoskeletal imaging, PET-MR image analysis, Open-source MSK imaging pipeline

## Abstract

**Objective:**

To develop and validate a fully automated, extendable, open-source AI-driven pipeline capable of knee magnetic resonance imaging (MRI) segmentation and quantitative extraction of MRI and positron emission tomography (PET) imaging biomarkers.

**Design:**

For model training, open-source knee MRI data from 347 knees acquired across 7 scanners using multiple gradient-echo sequences were used. An independent test set of 20 knees from 3T PET-MRI examinations were used for evaluation in this prospective reliability and agreement study. The pipeline segmented femoral, tibial, and patellar bones and cartilage, and medial and lateral menisci using three different deep learning models. Quantitative outputs included cartilage thickness and T_2_ relaxation times, meniscus volume and T_2_∗ relaxation times, bone shape scores, and static and kinetic subchondral bone PET measures. Manual segmentations from two annotators served as reference standards. Segmentation performance was assessed using Dice similarity coefficients (DSC), average symmetric surface distances, and volume differences. Quantitative outcome agreement was evaluated using intraclass correlation coefficients (ICC), Pearson correlations, and root mean square error.

**Results:**

Automated segmentations demonstrated high spatial agreement with manual annotations, with DSCs ranging from 0.84 to 0.98 across all tissues. Quantitative PET-MRI biomarkers generally demonstrated high agreement, although confidence intervals were wide for several biomarkers.

**Conclusion:**

OpenMSK enables multi-tissue knee segmentation and generation of quantitative MRI and PET biomarkers, with performance assessed against two independent annotators and across different U-Net-based segmentation architectures. As a freely available, modular platform, OpenMSK lowers technical barriers to whole-joint quantitative imaging and provides a standardized foundation for OA research and clinical translation.

## 1 Introduction

Osteoarthritis (OA) is a whole-joint disease involving the structural degeneration of multiple musculoskeletal tissues, including cartilage, meniscus, bone, and ligaments [1]. Understanding disease mechanisms and monitoring progression therefore requires quantitative assessment of these tissues, and is of paramount interest for basic science, clinical trials, and clinical decision making. MRI (MRI) is uniquely suited for this purpose, as it enables comprehensive morphological and compositional evaluation of the entire joint.

MRI-derived cartilage thickness change and T_2_ relaxation times are predictive of OA disease progression [2], [3], [4]. Cartilage thickness is a sensitive marker of early structural change [2], preceding radiographic OA. Elevated cartilage T_2_ signals collagen disruption and increased hydration, indicating compositional degeneration [3], [4]. Likewise, meniscal volume [5], [6] and T_2_∗ relaxation times [7], [8] quantify early meniscus degeneration, a known risk factor for OA.

Similarly, bone plays a central role in OA, contributing to structural degeneration, pain, and altered joint mechanics. MRI-based shape models [9] can quantify osteophytes, measure OA severity more precisely than the current diagnostic gold standard, radiographic Kellgren-Lawrence (KL) grades [10], and predict knee pain [9], [11], [12]. Furthermore, [^18^F]sodium fluoride positron emission tomography ([^18^F]NaF PET) imaging can evaluate bone metabolic activity, with studies demonstrating elevated tracer uptake in individuals with knee OA [13], [14].

While MRI- and PET-based quantitative measures provide a comprehensive framework for studying joint health, their application in research and translation to clinical practice remain limited for several reasons. First, manual or semi-automated segmentation is time consuming and susceptible to inter- and intra-user variability [15]. Second, when segmentations are available, extracting quantitative biomarkers requires substantial technical expertise, including quantitative MRI parameter map fitting [16], image registration [17], surface extraction and geometry processing for cartilage thickness calculation [18], and statistical shape modeling for bone-shape analysis [19]. Furthermore, most semi- or fully automated methods focus primarily on segmentation accuracy and often benchmark performance against a single manual annotator. Consequently, to accelerate research and enable clinical adoption of whole-joint quantitative imaging, a rigorously validated, modular, and open-source framework for comprehensive multi-tissue analysis is needed.

To address this need, we developed and validated a fully automated artificial intelligence (AI)-powered pipeline for comprehensive segmentation and quantitative analysis of multiple knee tissues from multi-modal MR and positron emission tomography (PET) images. In this work, we implemented and shared three models: one 2D convolutional neural network and two 3D nnU-Net [20] models. Using these models, we performed comprehensive internal validation of segmentations for major knee tissues (cartilage, bone, and meniscus) and evaluated downstream quantitative biomarkers (cartilage thickness and T_2_, meniscus volume and T_2_∗, neural shape model-based bone score, and [^18^F]NaF PET-derived measures of subchondral bone metabolism). Performance was assessed by comparing segmentations and derived quantitative metrics across three automated models and two manual annotators. The complete infrastructure is shared as a unified, open-source pipeline, ‘OpenMSK’ (https://github.com/gattia/OpenMSK) [21], and is freely accessible at http://www.openmsk.com/.

## 2 Methods

### 2.1 Image datasets for model training

Fat-suppressed gradient echo images are the standard for quantitative knee MRI analysis [22], [23]. We retrospectively curated a dataset of 347 knee volumes from previously reported studies: 176 standard double-echo steady state (DESS) image volumes from Siemens 3T scanners across four sites in the Osteoarthritis Initiative [24] (publicly available), 155 quantitative DESS (qDESS) image volumes from two 3T GE MR750 scanners [25] (publicly available), and 16 qDESS image volumes from subjects with known anterior cruciate ligament reconstruction from a 3T Siemens Magnetom [7] (all scan parameters for the training datasets are included in Supplementary Text S1). All training datasets contained manual segmentations for the same ten anatomical structures (femur, tibia, patella, femoral cartilage, medial tibial cartilage, lateral tibial cartilage, patellar cartilage, medial meniscus, and lateral meniscus). These datasets were used exclusively for model development and training, and all model evaluations were performed on newly acquired data. Quantitative DESS scans produce two echoes that can be combined for segmentation and can also be used to compute voxelwise T_2_ maps [22], [26].

### 2.2 Prospective imaging study

10 subjects with self-reported, symptomatic knee OA and 10 asymptomatic controls were recruited from the community as part of the Stanford Knee Osteoarthritis PET/MRI Evaluation (SKOPE) study [27] and prospectively imaged on a 3T GE Signa PET-MRI scanner (GE Healthcare, Waukesha, WI). The cohort size (n = 20) was selected as a feasibility sample for a reliability/agreement study rather than to estimate clinical performance. All participants gave written informed consent, and the study was conducted in accordance with the Institutional Review Board and was HIPAA-compliant. The qDESS MRI scan parameters are included in Supplementary Text S2. While only qDESS scans are required for tissue segmentation and the calculation of morphological and cartilage T_2_ metrics, this study also included additional image datasets. Dynamic [^18^F]NaF PET scans were acquired to calculate bone metabolism, perfusion, and mineralization for all 20 participants (protocol in Supplementary Text S3*)*, while multi-echo ultrashort echo time (UTE) MRI scans were acquired for meniscal T_2_∗ relaxometry for 15 out of 20 participants (scan parameters in Supplementary Text S4*)*. All the data is publicly shared at https://huggingface.co/datasets/agoyal5/OpenMSK_Validation.

### 2.3 Automated segmentation

We trained three models to segment three bones (femur, tibia, patella), four cartilage volumes (femoral, medial and lateral tibial, and patellar), and two meniscal volumes (medial and lateral).

#### 2.3.1 Model 1: 2D convolutional neural network (2D-CNN)

We used a 2D U-Net architecture as previously described [24] with an input size of 512 × 512, deep supervision (where deep layers are connected directly to the final output layer [28]), and batch normalization. Data augmentation included in-plane rotations (±6°) and translations (±20%); data augmentation was used as a standard component of model training to improve robustness and generalization and was not evaluated separately, consistent with prior work [20], [29]. The negative of the sum of individual tissue Dice similarity coefficients (DSC) was optimized using the Adam optimizer [30], batch size of 12, learning rate of 1e-4.5, dropout (0.2) and early stopping when improvement was <0.001 over 10 epochs. The network was implemented and trained using Keras 2 [31].

#### 2.3.2 Models 2 and 3: 3D nnU-Net- FullRes and Cascade

We trained nnU-Net [20] models as they represent an established and highly competitive baseline for medical image segmentation. We trained a 3D-FullRes network and a 3D-Cascade network using default training parameters. The FullRes segments 3D patches of the image in full resolution. Cascade first segments a low-resolution version and passes the output as an additional input to the same 3D-FullRes network.

### 2.4 Division of segmentations into subregions

#### 2.4.1 Cartilage

Femoral cartilage subregions were generated fully automatically from the segmentation labels using a rule-based algorithm implemented within OpenMSK. The femoral cartilage was partitioned into five subregions (anterior, medial and lateral central (weight-bearing), medial and lateral posterior) using established region definitions [32], [33] (Fig. 1). All cartilage regions were subsequently divided into equal-depth deep and superficial layers using an automated surface-distance approach implemented within the framework [22], [25]. Additional implementation details are provided in Supplementary Text S5.

**Fig. 1:**
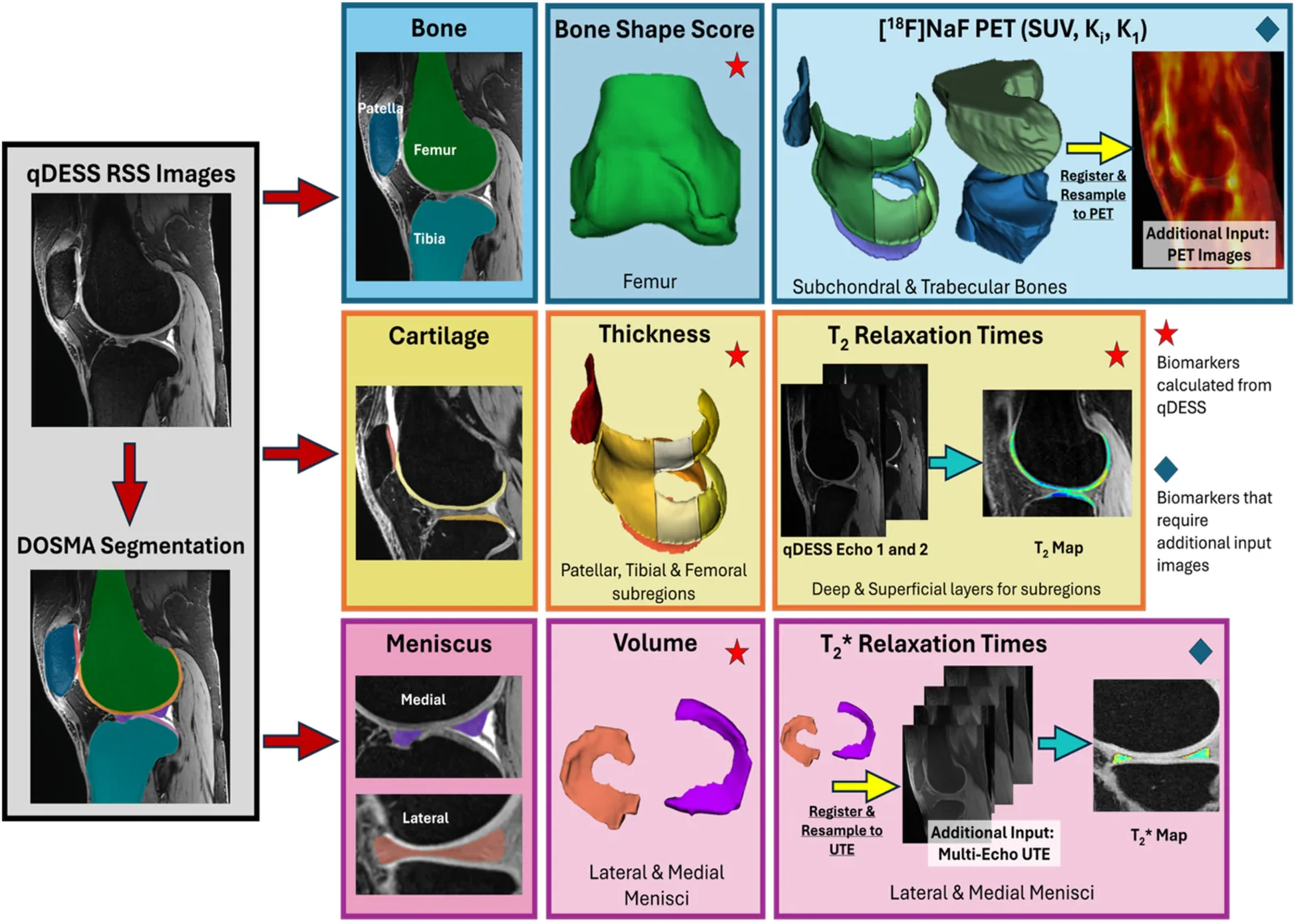
Overview of the OpenMSK pipeline [21], [34]. Using a qDESS volume as the reference input, the pipeline automatically segments knee bones (femur, tibia, patella), cartilage (femoral, patellar, medial and lateral tibial), and menisci (lateral and medial). These segmentations are automatically subdivided into anatomical regions and processed through tissue-specific analysis modules. Biomarkers marked with red stars are derived directly from the qDESS acquisition and include bone shape scores, cartilage thickness, cartilage T_2_ relaxation times, and meniscal volumes. Biomarkers marked with blue diamonds require additional imaging inputs: bone and cartilage segmentations are resampled to dynamic [^18^F]NaF PET space for quantification of SUV and kinetic measures (K_1_, K_i_), while meniscus segmentations are registered to UTE MRI for quantification of meniscal T_2_∗ relaxation times.

#### 2.4.2 Bone

Subchondral and trabecular bone subregions were generated for [^18^F]PET analysis from whole bone segmentations (Fig. 1).

Subchondral bone (femur, tibia, patella) regions included all bone voxels within 3 mm of the overlying cartilage. This thickness threshold was chosen to account for the low resolution of [^18^F]NaF on typical PET systems (intrinsic: 4.05–6.08 mm isotropic [35], our system's reconstruction: 1.3 × 1.3 × 2.78 mm^3^) and to capture signal spill in PET images. Subchondral bone regions were sub-divided into the same anatomical regions as the cartilage.

Trabecular bone (femur, tibia) subregions were created by labeling all bone voxels greater than 9 mm from the bone surface (based on previous region definitions [36]), truncated at the epiphysis for both bones. More details about the segmentation technique are included in Supplementary Text S6.

### 2.5 Clinical quantitative measurements

The following outlines the quantitative outcome extraction modules implemented in OpenMSK; analysis methods have been previously validated and are integrated into a single, open-source pipeline for standardized musculoskeletal MRI and PET analysis [21], [34] (Fig. 1). OpenMSK is implemented as a modular Python framework that integrates established open-source software components for musculoskeletal image analysis while allowing individual segmentation or analysis modules to be replaced or extended independently. Core functionality builds upon established open-source musculoskeletal imaging libraries, including *DOSMA* for quantitative MRI processing, *pyMSKT* for mesh-based morphometric analyses, *NSM* for neural shape modeling, and *dyNaFknee* for PET processing, together with standard scientific Python libraries.

#### 2.5.1 Pipeline modularity and supported imaging inputs

OpenMSK is designed to accommodate variable imaging inputs through a modular analysis workflow. Bone, cartilage, and meniscus segmentation and morphometric analyses are supported for fat-suppressed DESS acquisitions from multiple 3T scanners and vendors. Additional quantitative analyses are performed only when the corresponding imaging data are available. Specifically, quantitative T_2_ estimation requires qDESS acquisitions, [^18^F]NaF PET data are integrated to compute standardized uptake values (SUV) and pharmacokinetic metrics (K_1_ and K_i_), and multi-echo UTE MRI enables meniscal T_2_∗ mapping. Thus, although segmentation is compatible with any fat-suppressed gradient-echo sequence, additional quantitative analyses are performed only when the corresponding imaging modalities are available.

#### 2.5.2 Cartilage

1) Mean Thickness

Cartilage thickness was computed by converting the cartilage and bone segmentations into surface meshes. For each vertex on the bone surface, cartilage thickness was calculated as the distance along the local surface normal to the corresponding articular cartilage surface. Mean cartilage thickness for each anatomical subregion was obtained by averaging all vertex-wise thickness measurements within that region [11], [37]. Additional implementation details are provided in Supplementary Text S5.

2) T_2_ Relaxation Times

Cartilage T_2_ was computed from qDESS MRI scans for each voxel using an established analytical approach [18], [22]. Voxelwise T_2_ was averaged over each region for whole cartilage, as well as separately for deep and superficial layers [18], [25].

#### 2.5.3 Bone

1) BScore

A BScore is a normalized shape model-derived measure of OA severity: a higher score indicating a bone more characteristic of OA [9], [19]. We output BScores using a previously established methodology [9] using our open-source neural shape model trained on 6325 knees from the Osteoarthritis Initiative [19].

2) Subchondral Bone Metabolism from [^18^F]NaF PET Imaging

[^18^F]NaF PET provides a quantitative assessment of bone remodeling [14]. A PET image analysis pipeline, described in Supplementary Text S7, was used to compute PET SUV, bone perfusion (K_1_) and total bone mineralization (K_i_) using the Hawkins two-compartment model [38]. SUV represents normalized tracer concentration and is a semi-quantitative measure of tracer uptake. K_1_ reflects perfusion, and K_i_ reflects ^18^F^−^ deposition into bone. Regional mean and maximum SUV, K_1_, and K_i_ were calculated.

#### 2.5.4 Meniscus

1) Volume

Medial and lateral meniscal volumes were computed as the product of the segmentation voxel count and voxel volume.

2) T_2_∗ Relaxation Times

T_2_∗ maps were computed from a mono-exponential curve-fit using four echoes from 3D radial UTE scans [7] (scan parameters in Supplementary Text S4). For region-based quantification, qDESS-derived meniscus segmentations were registered to the UTE volumes using Elastix [39], [40] and mean T_2_∗ was computed (additional information regarding image registration in Supplementary Text S8*)*.

### 2.6 Agreement between manual and automated segmentations

Two annotators with 1 and 2 years of experience manually segmented unilateral qDESS knee MRI scans (Section 2.2) from 20 subjects independently. To minimize the influence of annotator experience, all segmentations underwent quality control by a researcher with 5 years of experience, and any discrepancies were resolved through final review by a researcher with 10+ years of experience. Manual segmentations were analyzed using the same methods as the automated segmentations (Sections 2.4, 2.5).

#### 2.6.1 Comparison of segmentation quality

Dice similarity coefficients (DSC), average symmetric surface distances (ASSD), and volume differences (VD) were computed for each tissue using Numpy [41].

#### 2.6.2 Comparison of downstream quantitative metrics

Quantitative metrics were evaluated using intraclass correlation coefficients (ICC (2,1), two-way random-effects, single measurement, absolute agreement), root mean squared error (RMSE), Pearson's correlations (r), and Bland-Altman analysis (mean bias and 95% limits of agreement) for each quantitative biomarker using individual anatomical regions from each subject as the unit of analysis, along with 95% confidence intervals for ICC and Pearson's r. Pearson's correlation was used to quantify the strength of the linear association between measurements, whereas Bland-Altman analysis was used to evaluate systematic bias and limits of agreement between manual and automated measurements [42], [43]. Because confidence intervals provide information regarding the precision of agreement estimates, interpretation emphasized both ICC point estimates and their corresponding 95% confidence intervals rather than classification thresholds alone [44].

## 3 Results

### 3.1 Patient demographics

The validation cohort included 10 subjects with self-reported, symptomatic knee OA (5 male/5 female, 44 ± 17 years old [range 22–65 years], BMI 25.5 ± 4.4 kg/m^2^ [range 19.9–34.5 kg/m^2^]) and 10 asymptomatic adults (5 male/5 female, 54 ± 7 years old [range 42–69 years], BMI 26.6 ± 4.1 kg/m^2^ [range 20.9–34.7 kg/m^2^]). Two raters manually segmented all the data.

### 3.2 Automated segmentation performance

All three models demonstrated comparable performance (DSC, ASSD, and VD) relative to two manual annotators (Reader 1: R1 and Reader 2: R2). The 2D-CNN results are shown in the main manuscript as a representative example because the three evaluated models produced highly similar results; results from the 3D nnU-Nets are provided in the Supplementary Tables.

Image-based metrics comparing automated 2D-CNN segmentations with manual annotations from two readers are shown in Table 1 and Fig. 2. VD, and nnU-Net results are reported in Supplementary Table S1.

**Table 1:** Comparison of segmentation performance between manual ground truth segmentations (Reader 1: R1, Reader 2: R2) and automated segmentations (Auto, representing 2D-CNN model). Data are mean (standard deviation), and the best performing out of all three are shown in **bold**. ASSD: Average symmetric surface distance. Volume differences for all tissues, and the performance of femoral cartilage subregions and trabecular bones are included in Supplementary Table S1.

Region	Dice similarity coefficient (DSC)			ASSD (mm)		
	R1 vs Auto	R2 vs Auto	R1 vs R2	R1 vs Auto	R2 vs Auto	R1 vs R2
Whole bones						
Femur	0.98 (0.01)	0.97 (0.01)	**0.98 (0.01)**	0.26 (0.15)	0.32 (0.15)	**0.23 (0.08)**
Tibia	**0.98 (<0.01)**	0.97 (<0.01)	0.98 (<0.01)	**0.19 (0.03)**	0.22 (0.05)	0.19 (0.04)
Patella	0.95 (0.01)	0.95 (0.01)	**0.96 (0.01)**	0.25 (0.09)	0.25 (0.06)	**0.18 (0.05)**
Whole cartilage						
Femoral	**0.89 (0.02)**	0.87 (0.02)	0.86 (0.02)	**0.13 (0.05)**	0.16 (0.05)	0.17 (0.05)
Medial tibial	**0.87 (0.02)**	0.86 (0.03)	0.84 (0.03)	**0.13 (0.05)**	0.17 (0.08)	0.18 (0.06)
Lateral tibial	**0.89 (0.03)**	0.87 (0.03)	0.87 (0.03)	**0.17 (0.09)**	0.21 (0.09)	0.21 (0.07)
Patellar	**0.91 (0.02)**	0.88 (0.03)	0.89 (0.02)	**0.11 (0.06)**	0.16 (0.09)	0.16 (0.08)
Meniscus						
Medial	**0.88 (0.02)**	0.88 (0.03)	0.85 (0.03)	**0.20 (0.09)**	0.21 (0.09)	0.26 (0.08)
Lateral	**0.89 (0.02)**	0.87 (0.04)	0.85 (0.03)	**0.17 (0.05)**	0.27 (0.23)	0.30 (0.22)
Subchondral bones						
Anterior femoral	**0.90 (0.02)**	0.88 (0.02)	0.89 (0.02)	**0.18 (0.07)**	0.21 (0.08)	0.20 (0.07)
Medial central femoral	**0.87 (0.03)**	0.87 (0.03)	0.86 (0.04)	0.21 (0.10)	**0.20 (0.07)**	0.21 (0.12)
Lateral central femoral	0.89 (0.04)	**0.89 (0.03)**	0.87 (0.03)	0.19 (0.13)	**0.16 (0.07)**	0.20 (0.09)
Medial posterior femoral	**0.91 (0.02)**	0.88 (0.02)	0.88 (0.03)	**0.18 (0.12)**	0.22 (0.14)	0.21 (0.10)
Lateral posterior femoral	**0.90 (0.03)**	0.88 (0.03)	0.87 (0.03)	**0.16 (0.08)**	0.20 (0.09)	0.21 (0.09)
Medial tibial	**0.90 (0.02)**	0.88 (0.02)	0.88 (0.02)	**0.21 (0.15)**	0.23 (0.09)	0.26 (0.14)
Lateral tibial	**0.90 (0.03)**	0.87 (0.02)	0.88 (0.03)	**0.21 (0.12)**	0.28 (0.11)	0.25 (0.10)
Patellar	**0.92 (0.01)**	0.88 (0.03)	0.89 (0.03)	**0.09 (0.03)**	0.16 (0.06)	0.14 (0.06)

**Fig. 2:**
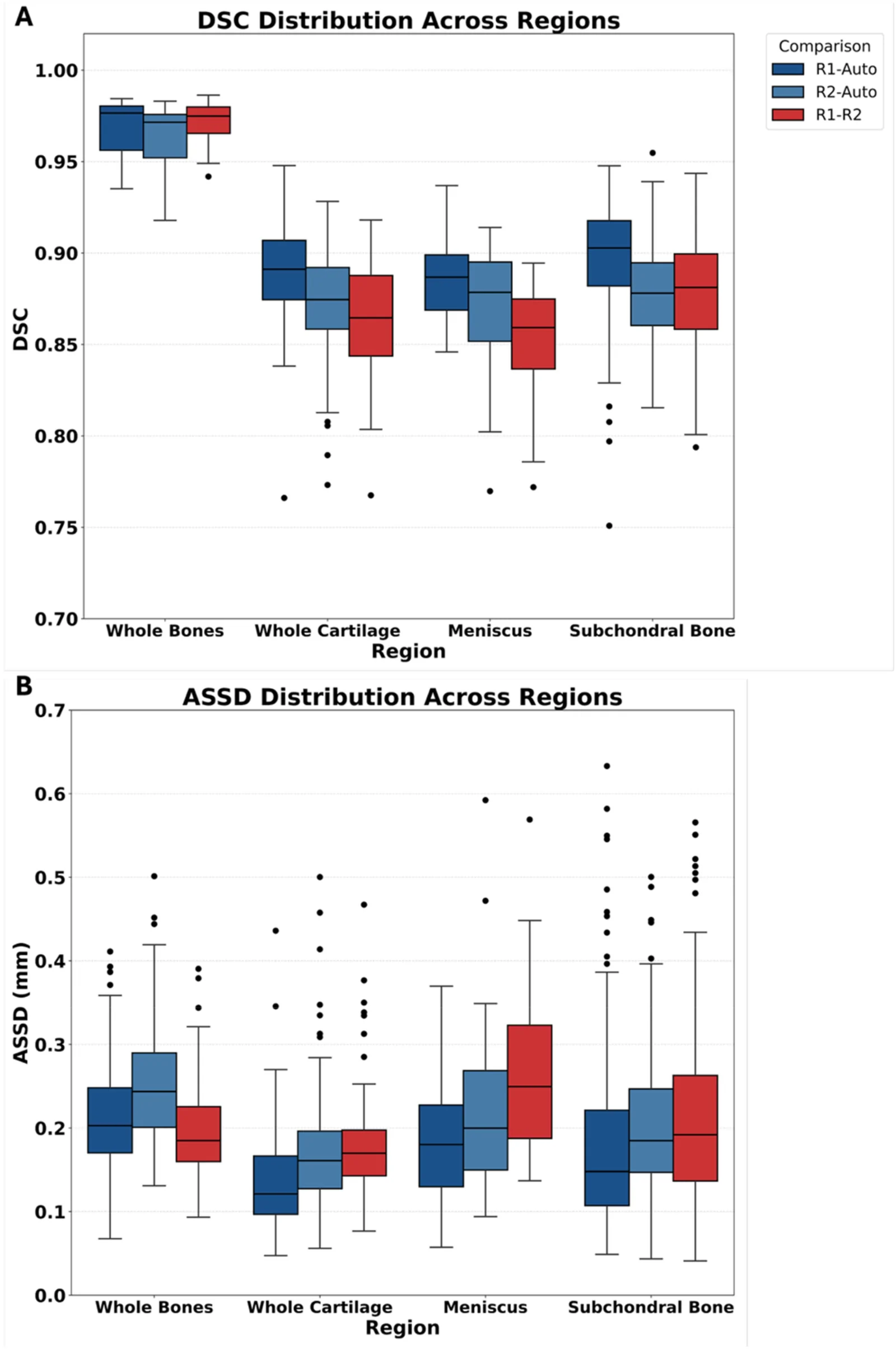
A) Dice similarity coefficient (DSC) and B) average symmetric surface distance (ASSD) across subjects and manual annotators (Readers) vs the 2D-CNN model for different regions of segmentations. Blue boxplots are manual versus automated, and red boxplots are inter-reader comparisons. R1: Reader 1, R2: Reader 2.

Whole Bones: The whole bones (femur, tibia, patella) show consistently high DSC (0.95–0.98). The ASSDs were less than in-plane resolution (0.19–0.32 mm), and VDs were positive (1.9–9.6%), indicating that the automated segmentations slightly overestimate volume.

Cartilage: Cartilage had worse DSCs (0.84–0.91) but better ASSDs (0.11–0.21 mm) than bone. The VDs were negative between manual and 2D-CNN segmentations (−5.8 to −9.6%), showing a tendency to underestimate volume compared to manual annotators.

Subchondral Bones: Subchondral bones exhibited high DSCs (0.86–0.92) and small ASSDs (0.14–0.28 mm), indicating good agreement.

Menisci: Lateral and medial menisci DSCs were in the range of 0.85–0.89 and ASSDs of 0.17–0.30 mm. VDs show larger variation, with regional averages for the lateral meniscus ranging from −10.7% to 1.7% and for the medial meniscus ranging from −3.7% to −1.6%.

### 3.3 Quantitative metrics

Downstream tissue-specific metrics were computed from two manual and three automated segmentations (Supplementary Table S2). Quantitative metric agreement between manual and automated segmentations from the 2D-CNN model for selected measures is shown in Table 2 (ICC, RMSE) and Fig. 3 (ICC, Pearson's r), with 95% confidence intervals reported for all the ICC and Pearson's r measurements. Compared to the 2D-CNN, nnU-Nets (Cascade and FullRes) produced nearly identical downstream agreement (ICC, RMSE, Pearson correlations, and Bland-Altman bias) compared to manual annotators; comprehensive comparisons are provided in Supplementary Table S3.

**Table 2:** Results of Intraclass correlation coefficient (ICC) analysis and root mean squared error (RMSE) analysis for quantitative metrics of cartilage, bone, and menisci, for the three comparisons between segmentation groups. Only some regions and quantitative metrics are included in-text, all results are included in Supplementary Table S3 along with Pearson correlation analysis. *Auto* represents the automated 2D-CNN segmentation model; results for the 3D models are in the Supplementary Table S3.

Region	ICC [mean (95% CI)]			RMSE [mean (range)]		
	R1 vs Auto	R2 vs Auto	R1 vs R2	R1 vs Auto	R2 vs Auto	R1 vs R2
**Bscore**						
Femur	0.96 (0.87, 0.99)	0.94 (0.34, 0.99)	0.97 (0.89, 0.99)	0.26 (0.09, 0.61)	0.32 (0.01, 0.71)	0.23 (0.02, 0.53)
**Meniscal volume**					**(mm^3^)**	
Medial meniscus	0.97 (0.86, 0.99)	0.97 (0.93, 0.99)	0.97 (0.92, 0.99)	150.49 (3.81, 278.61)	159.15 (7.62, 339.70)	173.79 (19.19, 524.71)
Lateral meniscus	0.93 (0.04, 0.99)	0.90 (0.02, 0.98)	0.98 (0.94, 0.99)	223.22 (13.18, 422.75)	274.47 (11.57, 500.10)	135.73 (32.67, 311.13)
**Cartilage thickness**					**(mm)**	
Medial central femoral	0.88 (0.62, 0.96)	0.80 (0.46, 0.93)	0.83 (0.62, 0.93)	0.13 (0.01, 0.29)	0.18 (0.01, 0.31)	0.17 (0.00, 0.38)
Patellar	0.91 (0.42, 0.97)	0.87 (−0.03, 0.97)	0.93 (0.79, 0.97)	0.20 (0.03, 0.40)	0.25 (0.15, 0.44)	0.17 (0.00, 0.37)
**Whole cartilage T_2_ relaxation times**					**(ms)**	
Medial central femoral	0.99 (0.96, 0.99)	0.93 (0.03, 0.99)	0.94 (0.24, 0.99)	0.73 (0.02, 1.93)	1.65 (0.33, 3.38)	1.49 (0.10, 2.70)
Patellar	0.98 (0.91, 0.99)	0.88 (−0.02, 0.97)	0.93 (0.04, 0.99)	0.57 (0.01, 1.55)	1.38 (0.25, 2.65)	1.02 (0.01, 1.97)
**SUV_mean_**						
Medial central femoral	1.00 (1.00, 1.00)	0.99 (0.98, 1.00)	1.00 (0.99, 1.00)	0.04 (0.00, 0.10)	0.04 (0.00, 0.12)	0.05 (0.00, 0.15)
Patellar	1.00 (1.00, 1.00)	1.00 (0.99, 1.00)	1.00 (1.00, 1.00)	0.01 (0.00, 0.03)	0.02 (0.00, 0.04)	0.02 (0.00, 0.04)
**K_i_: Total bone mineralization**					**(mL/min/mL x 10^−3^)**	
Medial central femoral	0.99 (0.98, 1.00)	0.99 (0.98, 1.00)	1.00 (0.99, 1.00)	4.19 (0.05, 10.69)	4.82 (0.01, 11.19)	4.46 (0.26, 11.14)
Patellar	1.00 (1.00, 1.00)	1.00 (0.99, 1.00)	1.00 (0.99, 1.00)	2.01 (0.16, 5.67)	2.57 (0.14, 6.40)	3.03 (0.02, 8.09)

**Fig. 3:**
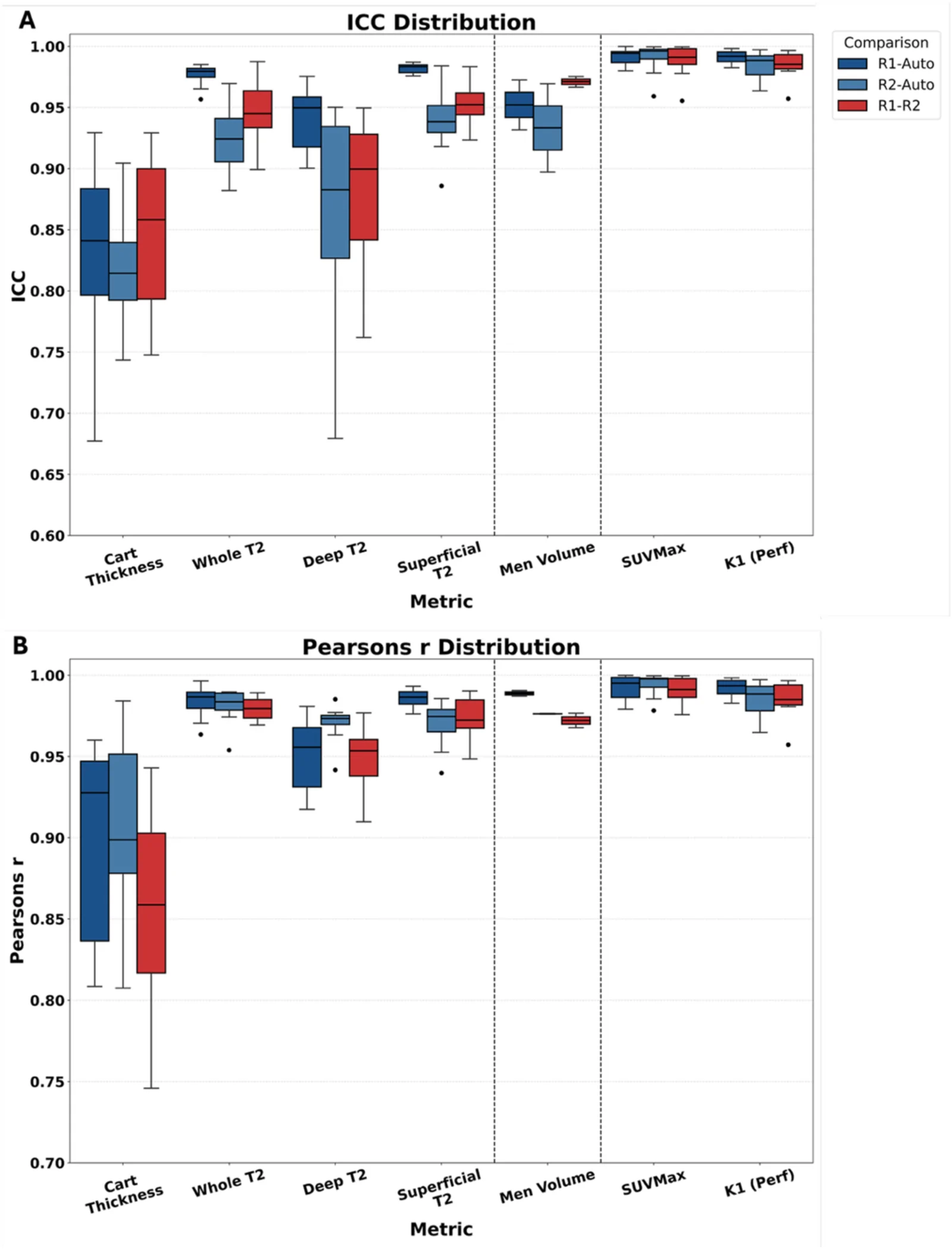
A) Intraclass correlation coefficients (ICC) and B) Pearson correlation coefficients (r) across subregions for selected quantitative metrics of cartilage, bone, and menisci, comparing both manual annotators (Readers) vs the 2D-CNN model. Cart: Cartilage, Men: Meniscus, K_1_ (Perf): Bone Perfusion, R1: Reader 1, R2: Reader 2.

#### 3.3.1 Cartilage

1) Mean Thickness

Across subregions, agreement between each reader (R1 or R2) and the automated method (2D-CNN) was generally high, with ICC point estimates ranging from 0.68 (medial tibial cartilage for R1 vs Auto) to 0.93 (medial posterior femoral cartilage for R1 vs Auto). Pearson's correlations ranged from 0.81 to 0.98, which indicates strong linear association between manual and automated measurements, and RMSE was <0.25 mm overall. Bland-Altman analysis demonstrated small mean biases with no consistent systematic over- or underestimation by the automated segmentations. Inter-reader agreement (R1 vs R2) was similarly high (ICCs 0.75–0.93, RMSE 0.10–0.17 mm, and Pearson's r 0.75–0.94). Although several ICC estimates had wide confidence intervals because of the modest sample size, point estimates and RMSE values were comparable between automated-versus-reader and inter-reader comparisons.

2) T_2_ Relaxation Times

When comparing metrics derived from the 2D-CNN vs manual segmentations, whole cartilage T_2_ had ICC point estimates between 0.96 and 0.99 (R1) and 0.89–0.97 (R2), and between 0.90 and 0.98 for inter-reader comparisons. Similarly, superficial cartilage had excellent agreement between readers (ICCs 0.89–0.99) while deep cartilage had more variability (ICCs 0.77–0.98). The poor deep cartilage ICCs were for R2 versus automated and R1 versus R2, and primarily in the patellar cartilage, while Bland–Altman analysis demonstrated small mean biases across T_2_ measurements, indicating no consistent systematic over- or underestimation. RMSE for whole cartilage T_2_ ranged from 0.52 to 2.5 ms for all comparisons. For all T_2_ metrics, the smallest errors were for R1 versus automated, followed by R1 versus R2, and finally R2 versus automated. For all regions except deep patellar cartilage, Pearson correlations were >0.9. Sample images showing segmented cartilage and T_2_ maps are in Fig. 4.

**Fig. 4:**
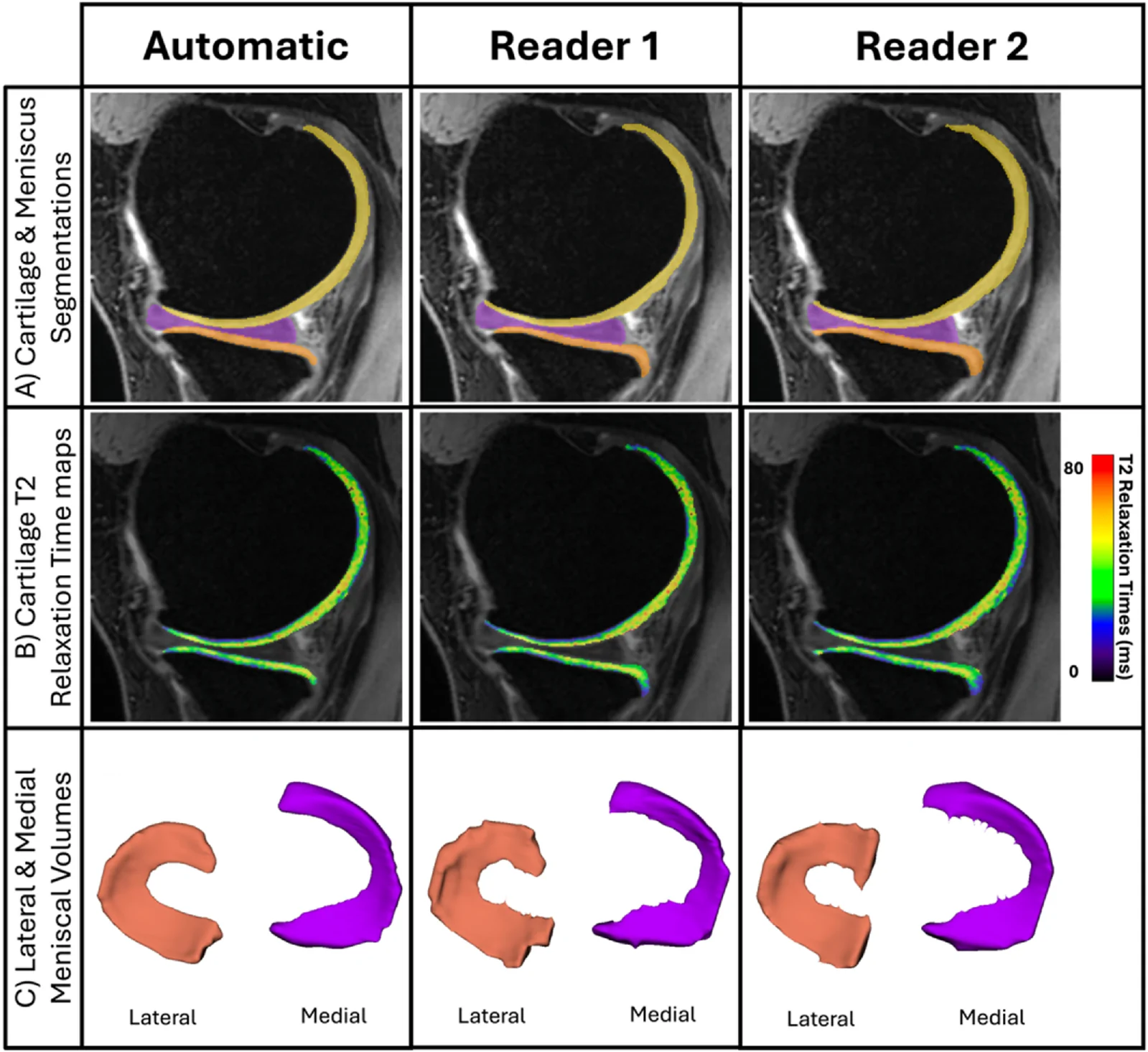
A) Sagittal MRI slices showing sample cartilage (femoral – yellow, lateral tibial – orange) and lateral meniscus (purple) segmentations generated by the automatic pipeline (2D-CNN), Reader 1, and Reader 2, for a subject in their fifties with self-reported knee osteoarthritis. B) T_2_ relaxation time maps overlaid on segmented cartilage demonstrate spatial agreement and consistency of quantitative mapping across methods. Color bar indicates T_2_ relaxation times in milliseconds (0–80 ms). C) 3D surface renderings of lateral (orange) and medial (purple) menisci, highlighting volumetric similarity and anatomical accuracy between the automatic method and both readers.

#### 3.3.2 Bone

1) BScore

High agreement was observed across all comparisons: ICC point estimates >0.94, Pearson's correlations >0.97, and RMSE from 0.23 to 0.32. However, confidence intervals for several ICC estimates were relatively broad, reflecting the limited precision expected from this feasibility reliability study. Bland–Altman analysis demonstrated small mean biases (−0.12 to 0.26) and narrow limits of agreement, indicating minimal systematic differences between manual and automated measurements. Fig. 5 shows variations in bone shape for a healthy control and a subject with OA.

2) Subchondral Bone SUV_mean_ and SUV_max_

**Fig. 5:**
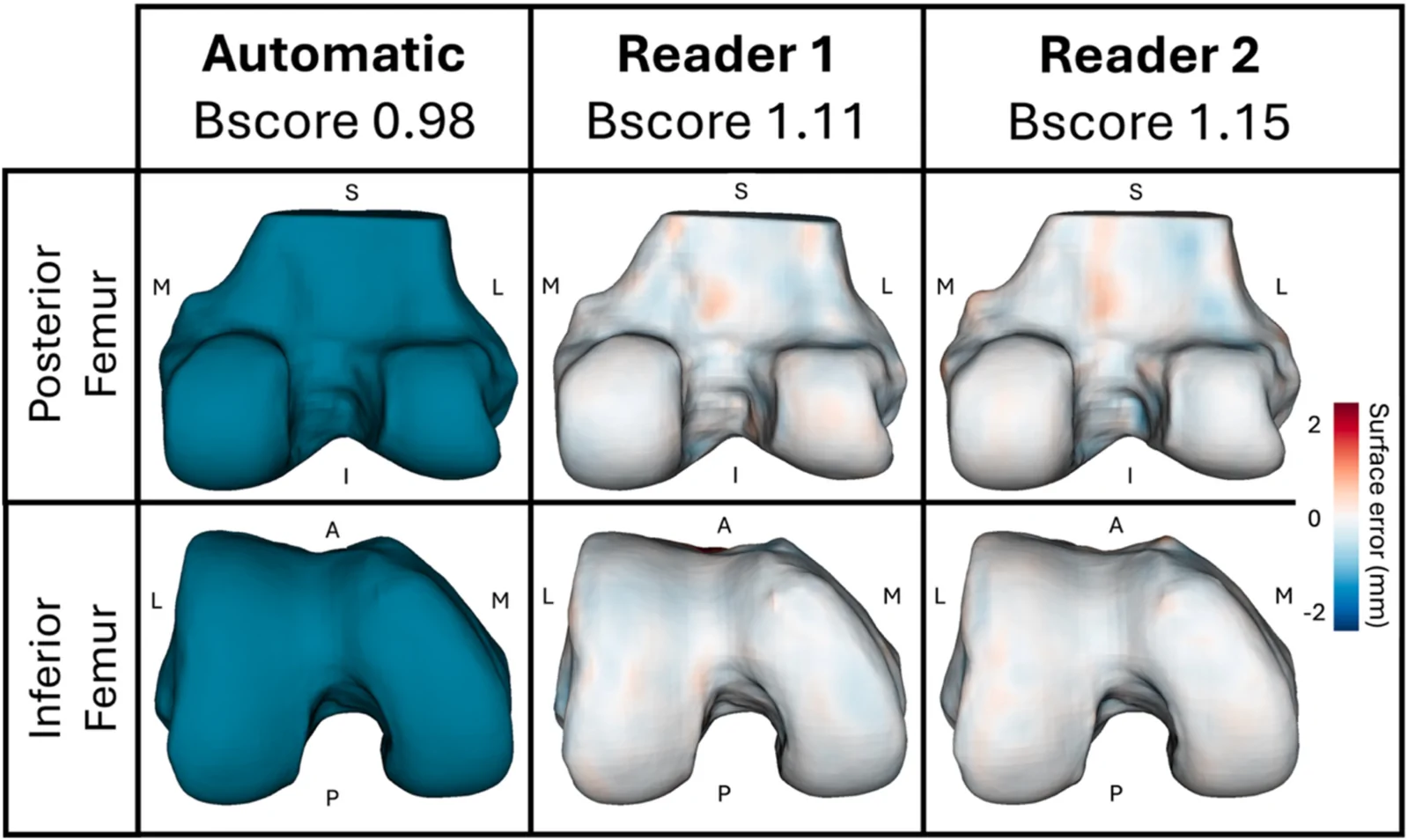
Bone shape models for a subject in their fifties with self-reported knee OA are shown, as the automatic segmentation meshes and the surface error models for the manual segmentations (as compared to the automatic segmentations from the 2D-CNN model). The colorbar represents the surface error between the automatic and manual meshes, with positive values showing that the manually segmented surfaces are outside the automatically segmented surface. The BScores for this subject range from 0.98 to 1.15. A mean BScore of 0 is equivalent to the average healthy bone from the training dataset, with 1 unit is equivalent to 1 standard deviation in the healthy group. All three segmentations produced BScores ∼1, congruent with a slightly non-healthy, i.e., OA bone shape.

ICC point estimates and Pearson's r for all comparisons were between 0.98 and 1.00. SUV_mean_ RMSE range 0.01–0.09 (for reference, mean: 0.95, range: 0.20–3.48) and SUVmax RMSE range 0.01–0.62 (for reference, ean: 3.01, range: 0.45–14.89). This indicates near-perfect consistency across manual and automated metrics, underscoring static PET analysis robustness.

3) Subchondral Bone K_1_ and K_i_

For both kinetic measures, all regions exhibited high agreement (ICC point estimates: 0.96–1.00) with low errors (RMSE: 2.00–4.97 × 10^−3^ mL/min/mL; for reference, K_1_: mean, range: 39.47, 3.09–141.59 × 10^−3^ mL/min/mL; K_i_: mean, range: 21.8, 3.05–73.94 × 10^−3^ mL/min/mL) and high Pearson's correlations (0.97–1.00).

#### 3.3.3 Meniscus

1) Volume

Measurements between R1 and R2 as well as R2 and the 2D-CNN showed high agreement, low RMSE and excellent correlations (ICC point estimates: 0.90–0.97, RMSE: 150–274 mm^3^, Pearson's r: > 0.98). Bland–Altman analysis demonstrated small mean biases with no evidence of consistent systematic over- or under-estimation between manual and automated measurements. For reference, mean volumes were 2437 mm^3^ while range was 1243–3785 mm^3^. Sample images showing segmented menisci and 3D volumes are in Fig. 4.

2) T_2_∗ Relaxation Times

Both readers and 2D-CNN showed high agreement for meniscus T_2_∗. For the medial meniscus, ICC point estimates were >0.98, with RMSE ≤ 0.27 ms (for reference, T_2_∗: mean: 9.12, range: 7.18–11.46 ms) and Pearson's r ≥ 0.99. For the lateral meniscus, ICC point estimates were >0.97, with RMSE ≤ 0.29 ms (for reference, T_2_∗: mean: 9.59, range: 7.53–12.04 ms), and Pearson's r ≥ 0.99. Bland–Altman analysis demonstrated minimal mean bias across all comparisons. Although point estimates were uniformly high, confidence intervals for several T_2_∗ measurements were wider because of the limited sample size.

## 4 Discussion

In this study, we developed and validated OpenMSK [21], [34], a modular, fully automated, open-source pipeline for comprehensive multi-tissue knee segmentation and quantitative MRI and PET analysis. Across the three evaluated U-Net-based deep learning models, automated segmentations of bones, cartilage, and menisci achieved performance comparable to inter-reader agreement and produced downstream quantitative biomarkers that closely matched those derived from manual segmentations. Importantly, quantitative measurements were highly consistent across the three models, indicating that the OpenMSK framework produces reproducible biomarker estimates for the segmentation models evaluated in this study. Overall segmentation performance was also highly similar, with only minor differences in DSC across tissues. Because of these comparable results and space constraints, the 2D-CNN results are presented in the main text as a representative example, while complete results for all three models are provided in the Supplementary Tables. Among the evaluated models, the 2D-CNN achieved the highest overall segmentation accuracy across tissues and is therefore recommended as the default model for most applications. The two 3D nnU-Net implementations produced comparable quantitative measurements and are provided as alternative implementations for users with different computational resources or workflow preferences. All trained models are publicly available, allowing researchers to select the implementation most appropriate for their application. By providing the complete pipeline as an open-source platform with both a user-friendly graphical interface [34] and a GitHub repository [21], OpenMSK aims to improve accessibility to advanced quantitative imaging tools and facilitate broader research applications and future clinical translation.

Automated segmentation performance was comparable to inter-reader variability across all tissues and metrics. DSC, ASSD, and VD demonstrated agreement within the range of previously reported benchmarks for knee bone [45], [46], cartilage [43], [45], [46], [47], and meniscus [43], [48], [49], [50] segmentations While inter-reader agreement varied across tissues and metrics, automated segmentations consistently fell within this variability range, indicating that model-derived segmentations are comparable to manual annotations. Performance was highly similar across the 2D-CNN and both 3D nnU-Net models, with consistent overlap, boundary, and volumetric metrics. For example, femoral cartilage DSC values were nearly identical across models and matched the range observed between manual readers. These findings align with results from the IWOAI knee segmentation challenge [43], which demonstrated that multiple deep learning architectures achieve similar segmentation performance when trained on comparable datasets.

Across cartilage, meniscus, bone shape, and PET, automated outputs generally showed moderate-to-excellent agreement with manually derived values. Bland-Altman analyses demonstrated minimal systematic bias between manual and automated measurements across biomarkers. While several ICC confidence intervals were broad because of the modest sample size, mean differences between methods remained small and limits of agreement were generally narrow relative to the physiological range of each quantitative biomarker. These findings suggest that disagreements were driven primarily by measurement variability rather than consistent over- or under-estimation by the automated segmentations. Specifically, cartilage thickness and T_2_ relaxation differences remained within established test–retest reproducibility limits (RMSE thickness < 0.25 mm, RMSE T_2_ < 2.5 ms) [50], [51], [52], [53], [54], [55]. Bone shape scores demonstrated small absolute errors relative to the observed cohort range (RMSE 0.23–0.32, range −0.97–2.64). Similarly, PET-derived SUV and kinetic parameters (K_1_, K_i_) extracted from automated subchondral bone regions were highly consistent with manual analyses (ICC > 0.96). This high agreement likely also reflects the relatively low spatial resolution of PET, which reduces sensitivity to small differences in segmentation boundaries. Across all tissues and modalities, the three segmentation architectures resulted in equivalent quantitative outputs, indicating derived metrics are largely architecture independent.

A central contribution of this work is the development of a modular, open-source, end-to-end framework that integrates segmentation, region definition, and quantitative analysis across MRI and PET imaging. The platform is freely available, user-friendly, and designed for extensibility, allowing substitution of segmentation models or adaptation to additional MRI sequences and PET tracers [56]. While several groups have developed tools for specific quantitative imaging tasks, many existing workflows remain proprietary, laboratory-specific, or focused mainly on segmentation without standardized downstream biomarker extraction [42], [45], [46], [47], [48], [49], [50], [51], [52]. In contrast, OpenMSK provides a unified infrastructure, from segmentation to validation of quantitative analysis across multiple tissues and imaging modalities. Importantly, biomarkers were highly consistent across three different deep learning models, indicating that the pipeline is largely agnostic to the segmentation models we tested. This allows researchers to integrate their own models (e.g., trained on different sequences or contrasts) while leveraging the same standardized framework to generate reproducible quantitative imaging outcomes, supporting scalable research and future clinical applications. In multimodal PET/MRI studies, the framework enables MRI-based segmentations to guide anatomically informed PET analyses, improving the localization and interpretation of PET tracers and biomarkers [56], [57].

### 4.1 Limitations

This study included a relatively small internal validation cohort for measuring reliability and agreement, with all data acquired on a single scanner; consequently, confidence intervals around several ICC estimates were broad and should be interpreted as reflecting limited precision rather than uncertainty regarding the observed point estimates. However, validation was strengthened by two manual annotations and inclusion of all major knee tissues. Although the released models were trained and evaluated on fat-suppressed DESS images acquired across multiple 3T scanners and vendors, their performance has only been validated within these imaging environments. Consequently, the current models should not be assumed to generalize to other sequence types (e.g., FSPGR) or field strengths (e.g., 1.5T) without additional validation. The pipeline's modular architecture, however, facilitates training and integration of models optimized for additional MRI sequences and imaging protocols. In addition, hybrid PET–MRI systems remain limited in availability, which may restrict the immediate applicability of the multimodal PET analysis components. However, to fully leverage these emerging imaging platforms, extract their scientific potential, advance understanding of the metabolic basis of joint disease, and enable clinical translation, standardized and validated analysis tools such as OpenMSK are essential.

## 5 Conclusion

OpenMSK provides a standardized, automated, and openly accessible framework for multi-tissue knee MRI and PET analysis. Segmentation performance falls within the range of manual variability, and downstream quantitative biomarkers are robust across the three U-Net-based segmentation models evaluated in this study. OpenMSK's modular, model-agnostic design enables integration of new segmentation approaches while supporting reproducible quantitative imaging and scalable research and clinical applications.

## Author contributions

All authors have made substantial contributions to various components of the study, drafting and revising the article, and approving the final version for submission.

**Ananya Goyal**: conceptualization, data curation, formal analysis, investigation, methodology, project administration, software, validation, visualization, writing – original draft preparation, writing – review and editing.

**Francesca Belibi**: investigation, methodology, validation, writing – review and editing.

**Vyoma Sahani**: investigation, methodology, validation, writing – review and editing.

**Rune Pedersen**: formal analysis, software, writing – review and editing.

**Yael Vainberg**: data curation, writing – review and editing.

**Ashley Williams**: data curation, methodology, writing – review and editing.

**Constance Chu**: data curation, funding acquisition, writing – review and editing.

**Bryan Haddock**: conceptualization, writing – review and editing.

**Garry E. Gold**: conceptualization, funding acquisition, resources, writing – review and editing.

**Akshay S. Chaudhari**: conceptualization, funding acquisition, resources, writing – original draft preparation, writing – review and editing.

**Feliks Kogan**: conceptualization, funding acquisition, resources, supervision, writing – original draft preparation, writing – original draft preparation, writing – review and editing.

**Anthony A. Gatti**: conceptualization, data curation, formal analysis, investigation, methodology, project administration, software, supervision, validation, visualization, writing – original draft preparation, writing – review and editing.

Ananya Goyal (agoyal5@stanford.edu), Anthony Gatti (aagatti@stanford.edu), and Feliks Kogan (fkogan@stanford.edu) take responsibility for the integrity of the work, from inception to finished article.

## Declaration of generative AI in scientific writing

Generative AI was not used in scientific writing, beyond checking grammar.

## Role of the funding source

Dr. Kogan receives research support from 10.13039/100000002NIH R01AR079431.

Ms. Goyal receives graduate fellowship support from the Wu Tsai Human Performance Alliance, the Stanford DARE Program and the Siebel Scholars program.

Dr. Gatti receives postdoctoral fellowship support from the Wu Tsai Human Performance Alliance.

Dr. Chaudhari receives research funding from NIH grants R01 HL167974, R01HL169345, R01 AR077604, R01 EB002524, R01 AR079431, P41 EB027060; ARPA-H grants AY2AX000045 and 1AYSAX0000024-01; and NIH contracts 75N92020C00008 and 75N92020C00021.

Dr. Chu receives research support from NIH grant AR052784, DOD W81XWH-18-1-0590, VA RX- 002452, and VA CX-002455.

The study sponsors (grant sources) were not involved in the study design, writing or manuscript submission.

## Competing interests

Dr. Kogan receives research support from GE Healthcare.

Dr. Gatti is a shareholder of NeuralSeg, NodeAI, and GeminiOV.

Dr. Chaudhari is a co-founder of Cognita, holds equity interest in Cognita, Subtle Medical, LVIS Corp, and Brain Key, and provides consulting services to Patient Square Capital, Chondrometrics GmbH, and Elucid Bioimaging.

All other authors declare no conflict of interest.

Only Dr. Kogan, Dr. Gatti, and Ms. Goyal had control of the study data.

## Figures and Tables

**figs1:**
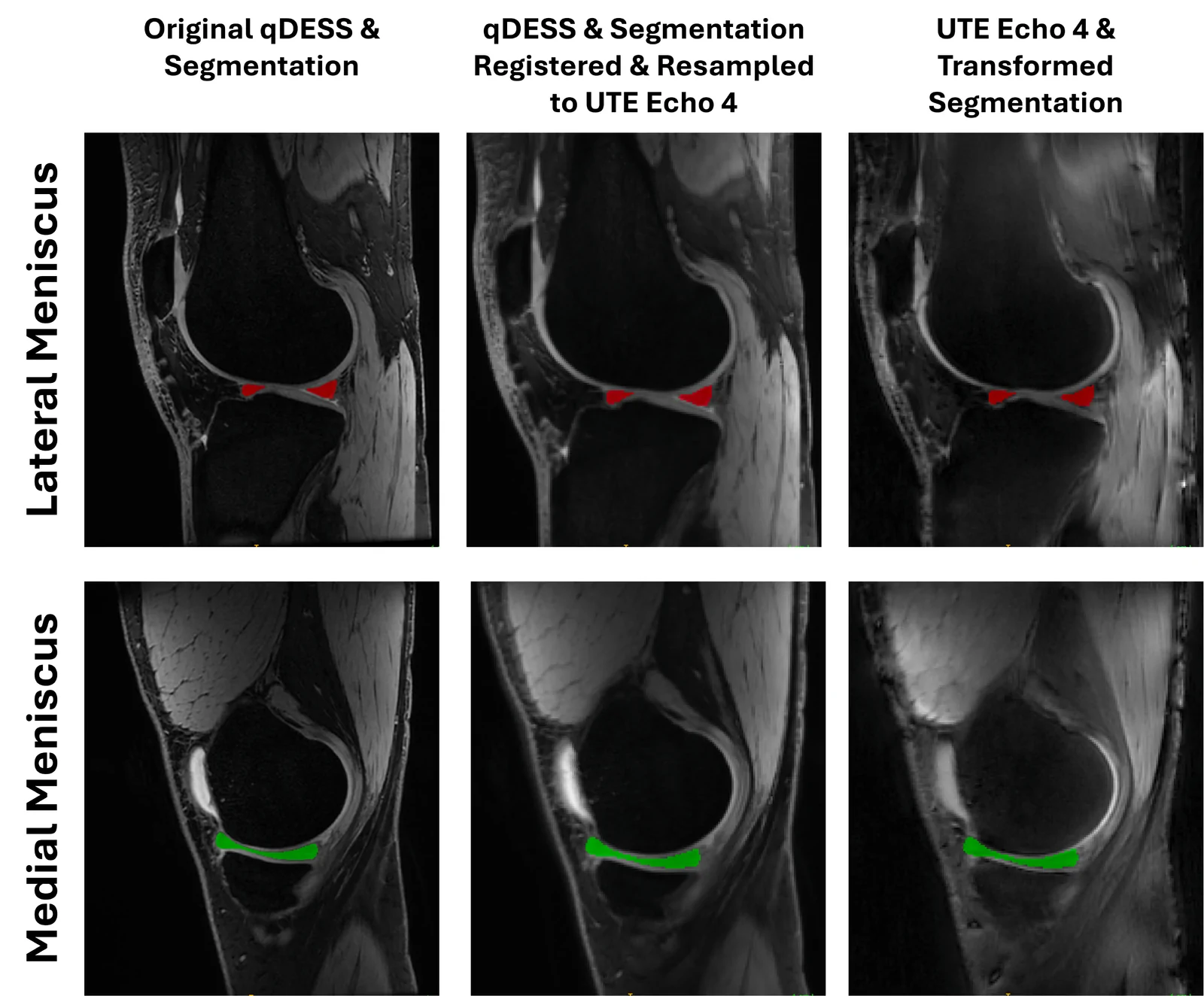


## References

[1] Coaccioli S., Sarzi-Puttini P., Zis P., Rinonapoli G., Varrassi G. (2022 Oct 12). Osteoarthritis: New Insight on Its Pathophysiology. JCM.

[2] Eckstein F., Collins J.E., Nevitt M.C., Lynch J.A., Kraus V.B., Katz J.N. (2015 Dec). Brief report: cartilage thickness change as an imaging biomarker of knee Osteoarthritis progression: data from the foundation for the national Institutes of Health Osteoarthritis biomarkers consortium. Arthritis Rheumatol..

[3] Atkinson H.F., Birmingham T.B., Moyer R.F., Yacoub D., Kanko L.E., Bryant D.M. (2019 Dec). MRI T2 and T1ρ relaxation in patients at risk for knee osteoarthritis: a systematic review and meta-analysis. BMC Muscoskelet. Disord..

[4] Prasad A.P., Nardo L., Schooler J., Joseph G.B., Link T.M. (2013 Jan). T1ρ and T2 relaxation times predict progression of knee osteoarthritis. Osteoarthr. Cartil..

[5] Xu D., Van Der Voet J., Hansson N.M., Klein S., Oei E.H.G., Wagner F. (2021 Mar 2). Association between meniscal volume and development of knee osteoarthritis. Rheumatology.

[6] Xu D., Schiphof D., Hirvasniemi J., Klein S., Oei E.H.G., Bierma-Zeinstra S. (2023 Dec). Factors associated with meniscus volume in knees free of degenerative features. Osteoarthr. Cartil..

[7] Williams A., Qian Y., Golla S., Chu C.R. (2012 Jun). UTE-T2⁎ mapping detects sub-clinical meniscus injury after anterior cruciate ligament tear. Osteoarthr. Cartil..

[8] Chu C.R., Williams A.A., West R.V., Qian Y., Fu F.H., Do B.H. (2014 Aug). Quantitative magnetic resonance imaging UTE-T2∗ mapping of cartilage and meniscus healing after anatomic anterior cruciate ligament reconstruction. Am. J. Sports Med..

[9] Bowes M.A., Kacena K., Alabas O.A., Brett A.D., Dube B., Bodick N. (2021 Apr). Machine-learning, MRI bone shape and important clinical outcomes in osteoarthritis: data from the Osteoarthritis Initiative. Ann. Rheum. Dis..

[10] Kellgren J.H., Lawrence J.S. (1957 Dec). Radiological assessment of osteo-arthrosis. Ann. Rheum. Dis..

[11] Gatti A.A., Keir P.J., Noseworthy M.D., Maly M.R. (2022 Mar 14). Investigating acute changes in osteoarthritic cartilage by integrating biomechanics and statistical shape models of bone: data from the osteoarthritis initiative. Magn Reson Mater Phy.

[12] Gatti A.A., Kogan F., Delp S.L., Gold G.E., Chaudhari A.S. (2023 Mar). Predicting chronic knee pain using an automated mri-based bone and cartilage statistical shape model: data from the Osteoarthritis initiative. Osteoarthr. Cartil..

[13] Kogan F., Fan A.P., McWalter E.J., Oei E.H.G., Quon A., Gold G.E. (2017 Jun). PET/MRI of metabolic activity in osteoarthritis: a feasibility study. Magn. Reson. Imaging.

[14] Goyal A., Watkins L., Bruce O., Gatti A., Kogan F. (2024 Sep). Metabolic bone imaging and its relationship with biomechanics. Osteoarthritis Imaging.

[15] Eckstein F., Kunz M., Schutzer M., Hudelmaier M., Jackson R.D., Yu J. (2007 Nov). Two year longitudinal change and test–retest-precision of knee cartilage morphology in a pilot study for the osteoarthritis initiative. Osteoarthr. Cartil..

[16] Eagle S., Potter H.G., Koff M.F. (2017 Mar). Morphologic and quantitative magnetic resonance imaging of knee articular cartilage for the assessment of post-traumatic osteoarthritis. J. Orthop. Res..

[17] Urish K.L., Williams A.A., Durkin J.R., Chu C.R., the OAI Investigators Group (2013 Jan). Registration of magnetic resonance image series for knee articular cartilage analysis: data from the Osteoarthritis initiative. Cartilage.

[18] Desai A, Barbieri M, Mazzoli V, Rubin E, Black MS, Watkins LE. DOSMA: a deep-learning, open-source framework for musculoskeletal MRI analysis. In: Proceedings of the 27th Annual Meeting of the International Society for Magnetic Resonance in Medicine (ISMRM) [Internet]. Montreal, QC, Canada. Available from: https://archive.ismrm.org/2019/1135.html.

[19] Gatti A.A., Blankemeier L., Van Veen D., Hargreaves B., Delp S.L., Gold G.E. (2025 Mar). ShapeMed-Knee: a dataset and neural shape model benchmark for modeling 3D femurs. IEEE Trans. Med. Imag..

[20] Isensee F., Jaeger P.F., Kohl S.A.A., Petersen J., Maier-Hein K.H. (2021 Feb). nnU-Net: a self-configuring method for deep learning-based biomedical image segmentation. Nat. Methods.

[21] Gatti A.A. (2025). https://github.com/gattia/OpenMSK.

[22] Sveinsson B., Chaudhari A.S., Gold G.E., Hargreaves B.A. (2017 May). A simple analytic method for estimating T2 in the knee from DESS. Magn. Reson. Imaging.

[23] Peterfy C.G., Schneider E., Nevitt M. (2008 Dec). The osteoarthritis initiative: report on the design rationale for the magnetic resonance imaging protocol for the knee. Osteoarthr. Cartil..

[24] Gatti A.A., Maly M.R. (2021 Dec). Automatic knee cartilage and bone segmentation using multi-stage convolutional neural networks: data from the osteoarthritis initiative. Magn Reson Mater Phy.

[25] Desai A.D., Schmidt A.M., Rubin E.B., Sandino C.M., Black M.S., Mazzoli V. (2022). SKM-TEA: a dataset for accelerated MRI reconstruction with dense image labels for quantitative clinical evaluation [Internet]. arXiv.

[26] Chaudhari A.S., Black M.S., Eijgenraam S., Wirth W., Maschek S., Sveinsson B. (2018 May). Five-minute knee MRI for simultaneous morphometry and T_2_ relaxometry of cartilage and meniscus and for semiquantitative radiological assessment using double-echo in steady-state at 3T. Magn. Reson. Imaging.

[27] Goyal A., Vainberg Y., Shalit R., Gatti A.A., Kogan F. (2026). The Stanford Knee Osteoarthritis PET/MRI Evaluation (SKOPE) Study protocol. Radiology and Imaging.

[28] Kayalibay B., Jensen G., van der Smagt P. (2017). CNN-based segmentation of medical imaging data. arXiv.

[29] Desai A.D., Gold G.E., Hargreaves B.A., Chaudhari A.S. (2019). Technical considerations for semantic segmentation in MRI using convolutional neural networks. arXiv.

[30] Kingma D.P., Ba J. (2014). Adam: a method for stochastic optimization [Internet]. arXiv.

[31] Chollet F. (2015). Keras.

[32] Eckstein F., Wirth W. (2011 Dec 8). Quantitative cartilage imaging in knee osteoarthritis. Arthritis.

[33] Panfilov E., Tiulpin A., Nieminen M.T., Saarakkala S., Casula V. (2022 May). Deep learning-based segmentation of knee MRI for fully automatic subregional morphological assessment of cartilage tissues: data from the Osteoarthritis Initiative. J. Orthop. Res..

[34] Gatti A.A. (2025). http://www.openmsk.com.

[35] Grant A.M., Deller T.W., Khalighi M.M., Maramraju S.H., Delso G., Levin C.S. (2016 Apr 13). NEMA NU 2-2012 performance studies for the SiPM-based ToF-PET component of the GE SIGNA PET/MR system: PET performance measurements of the GE SIGNA PET/MR. Med. Phys..

[36] Haddock B., Fan A.P., Jørgensen N.R., Suetta C., Gold G.E., Kogan F. (2019 May). Kinetic [18F]-Fluoride of the knee in normal volunteers. Clin. Nucl. Med..

[37] Ivanochko N.K., Gatti A.A., Stratford P.W., Maly M.R. (2024 Jul). Interactions of cumulative load with biomarkers of cartilage turnover predict knee cartilage change over 2 years: data from the osteoarthritis initiative. Clin. Rheumatol..

[38] Hawkins R.A., Choi Y., Huang S.C., Hoh C.K., Dahlbom M., Schiepers C. (1992 May). Evaluation of the skeletal kinetics of fluorine-18-fluoride ion with PET. J. Nucl. Med..

[39] Klein S., Staring M., Murphy K., Viergever M.A., Pluim J. (2010 Jan). Elastix: a toolbox for intensity-based medical image registration. IEEE Trans. Med. Imag..

[40] Shamonin D. (2013). Fast parallel image registration on CPU and GPU for diagnostic classification of Alzheimer's disease. Front. Neuroinf..

[41] Harris C.R., Millman K.J., Van Der Walt S.J., Gommers R., Virtanen P., Cournapeau D. (2020 Sep 17). Array programming with NumPy. Nature.

[42] Schneider E., Nevitt M., McCulloch C., Cicuttini F.M., Duryea J., Eckstein F. (2012 Aug). Equivalence and precision of knee cartilage morphometry between different segmentation teams, cartilage regions, and MR acquisitions. Osteoarthr. Cartil..

[43] Desai A.D., Caliva F., Iriondo C., Mortazi A., Jambawalikar S., Bagci U. (2021 May 1). The international workshop on osteoarthritis imaging knee MRI segmentation challenge: a multi-institute evaluation and analysis framework on a standardized dataset. Radiol. Artif. Intell..

[44] Koo T.K., Li M.Y. (2016 Jun). A Guideline of selecting and reporting intraclass correlation coefficients for reliability research. J. Chiropract. Med..

[45] Ambellan F., Tack A., Ehlke M., Zachow S. (2019 Feb). Automated segmentation of knee bone and cartilage combining statistical shape knowledge and convolutional neural networks: data from the Osteoarthritis Initiative. Med. Image Anal..

[46] Kessler D.A., MacKay J.W., McDonnell S.M., Janiczek R.L., Graves M.J., Kaggie J.D. (2022 Jun). Segmentation of knee MRI data with convolutional neural networks for semi-automated three-dimensional surface-based analysis of cartilage morphology and composition. Osteoarthritis Imaging.

[47] Schmidt A.M., Desai A.D., Watkins L.E., Crowder H.A., Black M.S., Mazzoli V. (2023 Apr). Generalizability of deep learning segmentation algorithms for automated assessment of cartilage morphology and MRI relaxometry. Magn. Reson. Imaging.

[48] Tack A., Mukhopadhyay A., Zachow S. (2018 May). Knee menisci segmentation using convolutional neural networks: data from the Osteoarthritis Initiative. Osteoarthr. Cartil..

[49] Gaj S., Yang M., Nakamura K., Li X. (2020 Jul). Automated cartilage and meniscus segmentation of knee MRI with conditional generative adversarial networks. Magn. Reson. Med..

[50] Norman B., Pedoia V., Majumdar S. (2018 Jul). Use of 2D U-Net convolutional neural networks for automated cartilage and meniscus segmentation of knee MR Imaging data to determine relaxometry and morphometry. Radiology.

[51] Eckstein F., Brisson N.M., Maschek S., Wisser A., Berenbaum F., Duda G.N. (2024 Jul). Clinical validation of fully automated laminar knee cartilage transverse relaxation time (T2) analysis in anterior cruciate ligament (ACL)-injured knees— on behalf of the osteoarthritis (OA)-Bio consortium. Quant. Imag. Med. Surg..

[52] Wirth W., Eckstein F., Kemnitz J., Baumgartner C.F., Konukoglu E., Fuerst D. (2021 Jun). Accuracy and longitudinal reproducibility of quantitative femorotibial cartilage measures derived from automated U-Net-based segmentation of two different MRI contrasts: data from the osteoarthritis initiative healthy reference cohort. Magn Reson Mater Phy.

[53] Wirth W., Maschek S., Wisser A., Eder J., Baumgartner C.F., Chaudhari A. (2025 Mar). Evaluation of an automated laminar cartilage T2 relaxation time analysis method in an early osteoarthritis model. Skelet. Radiol..

[54] Eckstein F., Walter-Rittel T.C., Chaudhari A.S., Brisson N.M., Maleitzke T., Duda G.N. (2024 Sep). The design of a sample rapid magnetic resonance imaging (MRI) acquisition protocol supporting assessment of multiple articular tissues and pathologies in knee osteoarthritis. Osteoarthritis and Cartilage Open.

[55] Williams A.A., Asay J.L., Asare D., Desai A.D., Gold G.E., Hargreaves B.A. (2025 Feb). Reproducibility of quantitative double-echo steady-state T_2_ mapping of knee cartilage. Magn. Reson. Imaging.

[56] Kogan F., Yoon D., Teeter M.G., Chaudhari A.J., Hales L., Barbieri M. (2024 Sep). Multimodal positron emission tomography (PET) imaging in non-oncologic musculoskeletal radiology. Skelet. Radiol..

[57] Kogan F., Watkins L.E., Goyal A. (2025 Jan). PET-MRI: the promise of multi-tissue imaging of early disease mechanisms in osteoarthritis. Osteoarthr. Cartil..

