# Supplementary material for: OpenMSK: A modular open-source framework for comprehensive analysis of imaging biomarkers in knee osteoarthritis
Source: Osteoarthr Cartil Open. 2026 Oct 9;8(4):100886. doi: 10.1016/j.ocarto.2026.100886 (PMC13682006; doi:10.1016/j.ocarto.2026.100886)
Supplement: Supplementary file 1 [file mmc2.docx]

**Supplementary Text**

**Section S1: DESS Scan Parameters for Model Training**

| **Scan Parameter** | **OAI, Gatti et al. [25]** | **SKM-TEA [26]** | **Williams, et al. [7]** |
| --- | --- | --- | --- |
| Sequence Name | DESS  (N=176 volumes) | Quantitative DESS (N=155 volumes) | DESS (N=16 volumes) |
| Fat suppression | Water Excitation | Water Excitation | Water Excitation |
| Scanner Details | 4 x 3T Siemens scanners | 2 x 3T GE MR750 scanners | 3T Siemens Magnetom scanner |
| Matrix Size | 384 x 384 | 416 x 512 | 307 x 384 |
| Resolution (mm^2^) | 0.37 x 0.37 | 0.38 x 0.31 | 0.37 x 0.46 |
| Slice Thickness (mm) | 0.7 | 0.8 | 0.7 |
| TE (ms) | 4.7, 27.9 | 5.7, 30.1 | 4.7, 27.9 |
| TR (ms) | 16.3 | 17.9 | 16.3 |
| Flip Angle (°) | 25 | 20 | 25 |
| Parallel Imaging/ Acceleration | - | 2 x 1 | - |
| Number of Slices | 160 | 80 to 88 | 160 |
| Coil Setup | Quadrature transmit-receive coil | 15 or 16 channels | 8 channels |

**Section S2: qDESS Scan Parameters for the Prospective Validation Study [28]**

3D sagittal qDESS MRI was acquired on a 3T GE Signa PET-MRI scanner with scan parameters: repetition time (TR) 18.24 ms, echo times (TEs) 6.04 ms and 30.44 ms, acquisition matrix size 320x320 (reconstructed 512x512), field of view 16 cm, slice thickness 1.5 mm, interpolated voxel size 0.3125x0.3125x1.5 mm^3^, acceleration 2 (phase) x 1 (slice), 118 slices, flip angle 20°, echo train length 1, bandwidth 162.8 Hz/pixel, number of averages 1, acquisition time 3:58 (both knees). A medium-sized flexible 16-channel receive-only phased-array coil was used for data acquisition per knee (Neocoil, Milwaukee, USA).

**Section S3: PET Image Dataset Scan Parameters**

Scan participants were hand-injected via an intravenous catheter with 92.5 MBq [^18^F]NaF (~2.5 mCi), and scanned in a 3T Signa PET-MR scanner. Dynamic PET list-mode data was acquired for both knees for 30 minutes. In addition, 2-point Dixon MRI was acquired for MR-based attenuation correction. From the list-mode data, images for the arterial input function (AIF), time activity curve (TAC), PET angiography (PETA), and static end of scan, were reconstructed. The reconstructed PET images had resolution 1.3x1.3x2.78 mm. For calculation of the image-derived arterial input function (AIF), dynamic PET frame times of 2 x 5 seconds, 20 x 1 seconds, 10 x 10 seconds, 10 x 30 seconds, 5 x 1 minutes, and 9 x 2 minutes were reconstructed using time-of-flight–ordered subset expectation maximization with 3 iterations with corrections for decay, attenuation, scatter, random, and dead time. Time activity curves (TAC) of bone PET uptake were determined using dynamic PET time frames of 6 x 10 seconds, 10 x 1 minutes, and 9 x 2 minutes with the same corrections. For the PET angiography images, static PETA images were reconstructed 15 seconds after the bolus delivery. Similarly, static end-of-scan images were reconstructed from the last five minutes of the dynamic scan, 25-30 minutes after the bolus delivery.

**Section S4: UTE Scan Parameters**

UTE imaging was introduced after the prospective study had commenced; therefore, UTE data were not available for the first five participants, resulting in UTE data for 15 of the 20 knees. UTE imaging was acquired with TR = 88 ms, a matrix size of 256 × 256, field of view of 16 cm, voxel size of 0.63 × 0.63 × 3 mm^3^, and 36–40 slices. Two echo-time protocols were used: TE = 0.028, 3.2, 6.4, and 9.6 ms in 10/15 cases, and TE = 0.028, 2.8, 5.6, and 8.4 ms in 5/15 cases.

For each case, a monoexponential decay model was fitted using the available echo times to generate a subject-specific T_2_* map. The five segmentation approaches (2 manual, 3 deep learning) were then applied to this same T_2_* map to derive regional T_2_* measurements. Thus, although two UTE acquisition protocols were used, all five segmentation-derived T_2_* measurements within a given case were calculated from the same underlying T_2_* map, enabling direct within-case comparison of the five approaches.

**Section S5: Cartilage Subregions, Layers, and Thickness Measurements**

Femoral subdivisions:

Femoral cartilage regionalization was performed directly from the segmentation masks using anatomical landmarks derived from the segmented tissues. The femoral cartilage was first separated into anterior, weight-bearing, and posterior regions using predefined anterior and posterior slice boundaries. The anterior boundary is the most anterior point of the intercondylar notch defined when the femoral cartilage is flattened along the inferior-superior axis. The posterior boundary is defined as 60% of the distance from the anterior boundary to the most posterior femoral bone point. Medial and lateral compartments were then distinguished using the trochlear notch and the relative positions of the segmented medial and lateral tibial cartilage. The resulting femoral regions comprised anterior, medial weight-bearing, lateral weight-bearing, medial posterior, and lateral posterior cartilage.

Deep and superficial layers:

Deep and superficial cartilage layers were generated automatically from the segmentation masks using a relative-depth algorithm implemented in pyMSKT. The articular and bone surfaces were extracted from the cartilage and bone segmentations. For each cartilage voxel, the Euclidean distance to the nearest bone surface and nearest articular surface was computed in three-dimensional space using signed distance fields. Relative cartilage depth was calculated by dividing each voxel's distance to the bone surface by the sum of its distances to the bone and articular surfaces. Voxels with a relative depth less than 0.5 were classified as deep cartilage, while those with a relative depth of 0.5 or greater were classified as superficial cartilage.

Cartilage thickness and regional assignment:

Cartilage thickness was computed using the bone and cartilage surface meshes. For each vertex on the bone surface, a vector was projected along the local surface normal. If the normal intersected the cartilage surface at two locations (the bone–cartilage interface and the articular surface), cartilage thickness was calculated as the Euclidean distance between these intersection points and assigned to the corresponding bone vertex. If two valid intersections were not identified, a thickness of zero was assigned.

Cartilage subregions were assigned using the same surface-normal projection. For each bone vertex with a valid cartilage intersection, 100 equally spaced points were sampled along the line connecting the bone–cartilage interface and articular surface. The anatomical cartilage label occurring most frequently along the sampled line was assigned to that bone vertex.

Regional thickness measurements were then obtained by averaging all thickness measurements within a given region of interest (anterior, medial and lateral central (weight-bearing), and medial and lateral posterior femoral cartilage regions, as well as the corresponding tibial and patellar regions.”

**Section S6: Subchondral and Trabecular Bone Segmentation**

Our definition of subchondral and trabecular bone differs from previous manually defined ROI approaches[36]. Rather than manually delineating trabecular compartments and separating compact from trabecular bone using k-means clustering, OpenMSK generates these regions fully automatically from the bone and cartilage segmentation masks. Subchondral bone was defined as bone within 3 mm of the overlying cartilage to account for the spatial resolution and partial-volume effects of clinical [^18^F]NaF PET, while trabecular bone was defined as bone located more than 9 mm from the bone surface, producing anatomically distinct compartments with minimal overlap. Although this approach improves reproducibility and scalability (due to automation), the resulting ROIs are not directly equivalent to previously reported manually defined compartments and should therefore be interpreted within the context of this automated framework.

**Section S7: PET Image Processing Pipeline**

In-house MATLAB codes were used for pharmacokinetic analysis, which is shared through an open-source GitHub repository, dyNaFKnee (<https://github.com/ananya-goyal/dyNaFknee>). Descriptions of each code and their corresponding function is below:

- Make_AIF.m: Dynamic 4D AIF images and 3D PETA images are used to segment blood vessels and extract arterial input function from segmented region. For segmentation, peak activity is identified, background noise is removed, and area under the curve and gradient analysis are computed to refine the vessel selection. Then, mean PET intensity values within the segmented vessel region are computed over time.
- TAC_coreg.m: Perform co-registration of 4D TAC (Time-Activity Curve) images to a single reference frame. We automatically segment the two legs and register them separately using monomodal rigid registration.
- Import_ROI.m: Load qDESS-based segmentations into the matrix.
- TAC.m: Loads the pre-defined ROIs and co-registered PET TAC data. Uses the function makeTACgraph to calculate TAC curves for the specified ROIs.
- ROI_seg.m: Loads AIF, TAC data, along with segmentations (ROIS) into the matrix. Divide ROIs into left and right regions for each scan.
- ROI_disp_kin.m: Using COMKAT, for each ROI, compute TAC, and apply a kinetic model. Fits a two-tissue compartment model to the TAC using the AIF and calculates parameters: rate constants (K_1_, k_2_, k_3_), blood flow delay and tissue uptake (K_i_, DV, etc.).

**Section S8: qDESS to UTE Image Registration Pipeline and UTE-T_2_* Analysis**

**Supplementary Figure 1**. Representative examples of DESS-to-UTE registration. The qDESS-derived meniscus segmentation (red and green) was transformed to the highest-echo UTE acquisition using rigid and affine registration with Elastix [39,40]. Visual inspection demonstrated good anatomical correspondence between the transformed segmentation and the UTE meniscus anatomy across representative subjects.

Limitations of this registration pipeline

Meniscal T_2_* quantification required registration of qDESS-derived segmentations to the UTE acquisition. Although both sequences were acquired during the same imaging session and registration was limited to rigid and affine transformations to preserve anatomy, differences in image contrast and subject positioning may introduce small alignment errors, particularly near tissue boundaries. Representative registration results are provided above. Future work could further improve correspondence through sequence-specific segmentation models or registration methods optimized for multimodal musculoskeletal MRI.
